# When the “heroes” “don’t feel cared for”: The migration and resignation of Philippine nurses amidst the COVID-19 pandemic

**DOI:** 10.7189/jogh.12.03011

**Published:** 2022-05-23

**Authors:** Rowalt Alibudbud

**Affiliations:** Department of Sociology and Behavioral Sciences, De La Salle University, Manila City, Philippines

As socio-economic activities re-open and societies re-emerge from the COVID-19 pandemic, collective and effective COVID-19 responses among nations must be sustained. For this to occur, the needs and challenges of health care workers as essential parts of health systems and as central actors of the collective COVID-19 response must be addressed. While previous discussions and reports have focused on health issues such as burnout and depression [1], it is also essential to look into their rights, freedoms, and living conditions, as these may not only affect their health and well-being, but also their decision to partake in societal COVID-19 responses.

In this regard, this paper centres on the importance of upholding the rights, freedoms, and just living conditions of health care workers as exemplified by the situation of Filipino nurses in the Philippine health care system amidst the COVID-19 pandemic. The world faced the pandemic with a global shortage of nurses of about 5.9 million [2]. Asia is among the regions with the lowest density of nurses in the world, despite having countries that largely supply nurses in other regions [2]. The Philippines alone supplied about 240 000 nurses to Organisation for Economic Co-operation and Development (OECD) countries with an outflow of 15 000 to 20 000 nurses per year. This made the Philippines the largest supplier of nurses to OECD countries [2]. The high nurse-to-patient ratio and low wages were among the common reasons for Filipino nurses to work in other countries [2]. While it gave rise to a global diaspora of Filipino nurses, it also resulted in a low number and unequal distribution of nurses in the Philippines [2]. This migration and resignation of Filipino nurses from the Philippine health care system may have accelerated during the pandemic.

## FILIPINO NURSES AMIDST THE COVID-19 PANDEMIC

One year into the pandemic, recent news reports in the Philippines highlighted that Filipino nurses are resigning to work abroad. In the first two to three weeks of October 2021 alone, it was noted that about 5% to 10% of nurses working in private hospitals have resigned [3]. In another 2021 news report, a hospital director in a city mentioned that their nursing staff had decreased from 200 to 63 over the past year [4]. Overall, about 40% of nurses in private hospitals have resigned since the pandemic began [3]. Thus, hospitals in the Philippines may be understaffed due to the dwindling number of nurses during the pandemic.

Among the commonly cited reasons for the resignation remained to be low wages. An entry-level nurse working in a public hospital starts with a monthly salary of about PHP33 575 (about US$670), while those working in private hospitals may start with as little as PHP8000 (about US$160) [4]. These wages may not be enough to cover the cost of living in the Philippines. For example, the estimated cost of living in Metro Manila, the largest Philippine metropolitan area, is PHP50 798 (about US$1080) [5]. Some of the nurses even go to work without benefits and hazard pay, despite the heightened health risks and threats during the pandemic [4].

**Figure:**
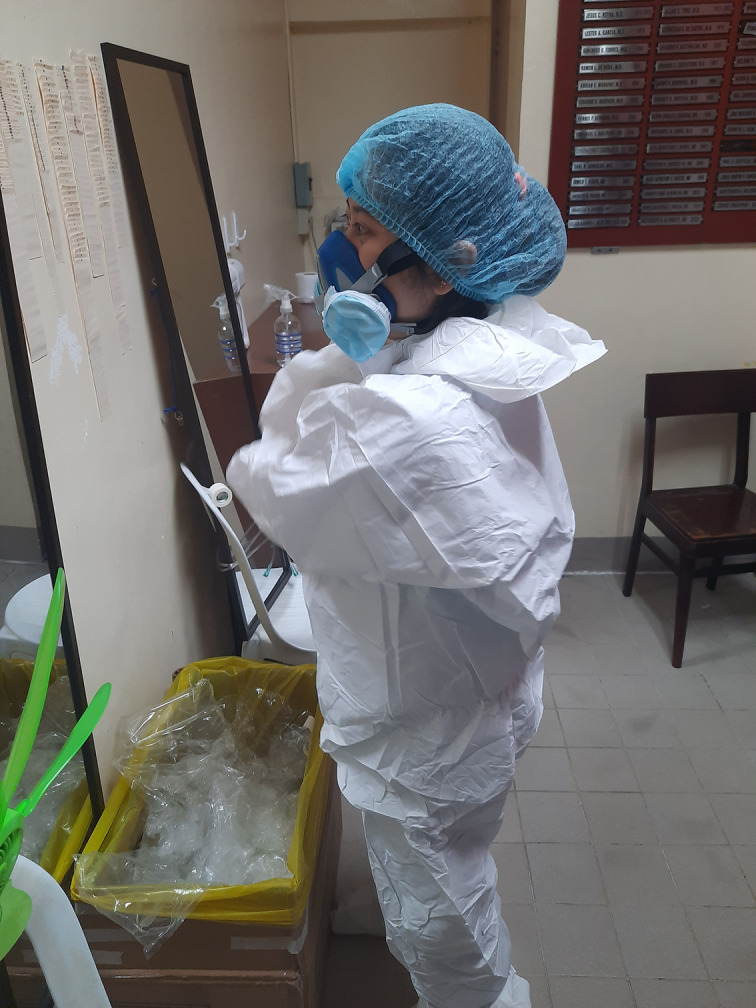
Photo: Filipino nurses’ daily routine during the COVID-19 pandemic (from Rowalt Alibudbud’s personal collection, used with permission).

## GOVERNMENT RESPONSES TO THE PLIGHT OF NURSES

Despite the need for livable wages and just benefits for Filipino nurses, the Philippine government responded by banning and limiting them from living and working abroad, so they could serve as a “reserve force” as the country navigates through the pandemic. This deployment ban was largely questioned due to its possible unconstitutionality, violation of the right to travel and earn a living wage and negative effect on the Philippine economy [6].

Nonetheless, some improvements have been done, such as the additional PHP500 (about US$10) daily allowance for health care workers who care for patients with COVID-19. However, its implementation has been met with confusion, dismay, and disappointment [7,8]. For instance, a 2020 news report showed that the daughter of a nurse who died from COVID-19 was appalled and dismayed when she claimed her mother’s hazard pay amounting to PHP7000 (about US$140) since she expected to receive PHP30 000 (about US$600) [7]. This was because the previously announced government daily allowance was reduced to PHP64 (about US$1.5) after it was adjusted for their city’s health budget and mandated deductions [7]. Amidst these news reports and the resignation of nurses, several health care worker groups have also highlighted that they were being forced to work long hours and had an inconsistent supply of personal protective equipment (PPE) [7].

A year after, the situation had seemingly remained the same, as disclosed by health care groups, with nurses forgoing their meals and bathroom breaks to save on PPEs. Moreover, it was reported that the promised additional compensation for health care workers had not been paid out. To them, “their working conditions are no longer humane” [8]. Thus, Filipino nurses seemed to be domestic captives in their own country. The barriers to escape are generally invisible and take form as economic, social, and legal subordination.

## RESIGNATION AND MIGRATION: SENTIMENTS AND RESPONSES OF FILIPINO NURSES

Given the chronic understaffing, low wages, unsafe working conditions, and deployment bans, Filipino nurses have expressed their exhaustion and dismay with statements such as “We don't feel cared for” and “We feel exhausted...but we always keep in mind that we have to help our people because...no one else will” [3,4]. Eventually, some of them may leave the profession or try to go abroad since “it's really not worth being a nurse at home” [4]. This seemed to be the sentiment of nurses and other health care worker groups who have announced their mass resignation from the Philippine health care system amidst the COVID-19 pandemic [8]. While some were able to migrate, remaining nurses in the Philippines, as seen in private hospitals [4], may leave their profession to escape their seeming domestic captivity and socio-economic hardships amidst the COVID-19 pandemic. Thus, Filipino nurses may be free when they no longer work as “nurses”.

## THE EFFECT OF THE RESIGNATION AND MIGRATION OF FILIPINO NURSES ON THE LOCAL COVID-19 RESPONSE

This flight of health care workers from health care institutions in the Philippines had severely affected the local COVID-19 response [3,4]. In 2021, hospitals in the country have already started to downsize their operations, not because of the lack of facilities or health equipment, but because of the lack of health care workers. Thus, despite the decreasing trends of COVID-19 in the country, hospitals remained fully occupied [3,4]. If allowed to worsen, the health care system may be overwhelmed by a new COVID-19 wave.

## HONOUR AND VALUE AS “HEROES”

Generally, while health care workers have been hailed as “heroes” in the recent pandemic [9], honour without just wages, adequate staffing, and livable conditions will not sustain the responses to COVID-19. Given this, governments, policymakers, and health care institutions must be ever cognizant of the rights and needs of nurses and other health care workers. If these are not addressed, health care workers, as exemplified by the resignation of Filipino nurses, may leave their profession and institutions to seek opportunities where their work is valued, and their rights are upheld. As a result, health care systems may collapse in the face of a tremendously challenging situation such as the COVID-19 pandemic. There is, therefore, a need to reflect the health care heroes’ honour and value in specific programs and policies implemented amidst the pandemic. Overall, the resignation and migration of Filipino nurses amidst the COVID-19 pandemic may not only be an issue related to health and well-being but also rights and justice. Nonetheless, it must be addressed.
